# Patient:innenbeteiligung in der Gesundheitsforschung in Deutschland: Gegenwärtiger Stand und Zukunftsperspektiven

**DOI:** 10.1007/s00103-025-04178-3

**Published:** 2026-01-14

**Authors:** Lena Oster, Avin Hell, Ines Moegling, Anna Levke Brütt

**Affiliations:** 1 Institut und Poliklinik für Medizinische Psychologie, Universitätsklinikum Hamburg-Eppendorf, Hamburg, Deutschland; 2 Hubertus Wald Tumorzentrum, Universitäres Cancer Center Hamburg (UCC Hamburg), Universitätsklinikum Hamburg-Eppendorf, Hamburg, Deutschland

**Keywords:** Bürger:innenbeteiligung, Versorgungsforschung, Partizipative Forschung, Patient:innenzentrierung, Patient and Public Involvement, Citizen participation, Health services research, Participatory research, Patient centricity, Patient and public involvement

## Abstract

Um Gesundheits- und Versorgungsforschung patient:innenzentriert zu gestalten, sollten die Sichtweisen und Erfahrungen der Patient:innen systematisch einbezogen werden. Patient:innenbeteiligung bedeutet, dass Betroffene nicht als Forschungssubjekt, sondern als Expert:innen mit Krankheits- und Versorgungserfahrungen zum Forschungsprozess beitragen. Wie dies zur Patient:innenzentrierung beiträgt, wird anhand dreier Aspekte beschrieben: Patient:innenbeteiligung kann sicherstellen, dass (1) relevante Forschungsthemen adressiert werden, (2) Prozesse für teilnehmende Personen passend gestaltet sind und (3) die Ergebnisse gemeinsam mit Patient:innen diskutiert werden.

In Deutschland wird die Umsetzung von Patient:innenbeteiligung durch Forschungsförderer und Patient:innenorganisationen forciert. Sowohl Patient:innen als auch Wissenschaftler:innen berichten von positiven Erfahrungen, aber auch von Herausforderungen. Forschungseinrichtungen können dafür nachhaltige Strukturen wie im Universitären Cancer Center Hamburg aufbauen, wo Patient:innen in Beiräten und als Botschafter:innen in der Krebsforschung mitwirken.

Für eine wirksame Umsetzung braucht es neben entsprechende Strukturen auch Wissen. Praxisnahe Leitfäden, Beispiele sowie Weiterbildungsangebote können hierfür eine Grundlage schaffen. Ebenso wichtig ist transparente Berichterstattung, um Beteiligungsprozesse kritisch zu reflektieren. Durch eine wissenschaftliche Evaluation lässt sich untersuchen, wie und unter welchen Bedingungen Patient:innen sinnvoll an Forschungsprojekten mitwirken können und welche Auswirkungen die Beteiligung auf Patient:innen, Wissenschaftler:innen, den Prozess und die Ergebnisse der Forschungsprojekte hat.

## Abstract (en)

In order to make health and healthcare research patient centred, the perspectives and experiences of patients should be systematically taken into account. Patient involvement means that those affected contribute to the research process not as research subjects, but as experts with lived experience of illness and healthcare. How this contributes to patient-centredness is described by means of three aspects: patient involvement can help to ensure that (1) relevant research topics are addressed, (2) research processes are designed to suit participants, and (3) results are discussed together with patients.

In Germany, research funders and patient organizations are promoting the implementation of patient involvement. Both patients and researchers report positive experiences but also challenges. Research institutions can establish sustainable structures for this, as exemplified by the University Cancer Center Hamburg, where patients participate in advisory boards and act as ambassadors in cancer research.

Effective implementation requires not only appropriate structures but also knowledge. Practical guidelines, examples, and training opportunities can provide a basis for this. Equally important is transparent reporting in order to critically reflect on involvement processes. Scientific evaluation can examine how and under which conditions patients can meaningfully contribute to research projects and what impacts involvement has on patients, researchers, and patient centeredness as well as on the process and the results of research projects.

## Hintergrund

Das Konzept der Patient:innenzentrierung findet nicht nur in der gesundheitlichen Versorgung Anwendung [1], sondern lässt sich auch auf die Gesundheitsforschung übertragen. Die Beteiligung von Bürger:innen und Patient:innen als aktiv Mitwirkende in Forschungsprojekten (englisch: patient and public involvement, PPI) stellt dabei eine zentrale Strategie dar, um sicherzustellen, dass Forschung den Bedürfnissen und Prioritäten der Nutzenden von Gesundheits- und Versorgungsleistungen entspricht [2]. Nicht nur die Forschung kann von dieser Beteiligung profitieren, auch Patient:innen selbst erfahren eine Anerkennung und Wertschätzung ihrer Erfahrungen. Gleichzeitig kann Patient:innenbeteiligung Wissenschaftler:innen tiefere Einsichten in die Relevanz ihres Forschungsthemas ermöglichen.

Patient:innenzentrierung bildet dabei zugleich die Grundlage und das Ziel von gelingender Patient:innenbeteiligung. Nur wenn Forschung die Bedürfnisse von Betroffenen berücksichtigt und auf diese eingeht, kann Patient:innenbeteiligung sinnvoll erfolgen. Umgekehrt trägt eine aktive Beteiligung dazu bei, Forschung und ihre Ergebnisse patient:innenzentrierter zu gestalten. Diese wechselseitige Beziehung steht im Zentrum der folgenden Ausführungen. Dabei wird auf 3 zentrale Aspekte der Beteiligung eingegangen, die eine patient:innenzentrierte Forschung ermöglichen. Am Beispiel des Universitären Cancer Centers Hamburg werden Strukturen für patient:innenzentrierte Forschung erläutert sowie die Zukunftsperspektiven dargestellt.

In Infobox 1 wird der in diesem Beitrag verwendete Begriff „Patient:innen“ im Bereich PPI eingeordnet. Denn im Kontext der Versorgungsforschung ist auch die Beteiligung weiterer Stakeholder wie Angehörige, medizinische Fachkräfte oder Akteure im öffentlichen Gesundheitssystem auch im Hinblick auf die Implementierung in der Versorgungspraxis von besonderer Bedeutung [3]. Welche Personen(gruppen) einbezogen werden, hängt vom jeweiligen Forschungsgegenstand ab.

## Patient:innenbeteiligung in der Gesundheits- und Versorgungsforschung

Um Gesundheits- und Versorgungsforschung patient:innenzentriert zu gestalten, sollten die Sichtweisen und Erfahrungen der Patient:innen in Forschungsprojekten systematisch einbezogen werden. Eine Möglichkeit besteht darin, dass Patient:innen in der Rolle der Versuchspersonen, Proband:innen oder Teilnehmenden verbleiben, etwa dann, wenn Interviews oder Fokusgruppen durchgeführt werden, um Bedürfnisse zu erheben, die dann im weiteren Forschungsprozess von den Wissenschaftler:innen berücksichtigt werden. In diesen Fällen sind Patient:innen Forschungssubjekte, also der Gegenstand der Forschung selbst.

Die aktive Mitwirkung im Forschungsprozess ist mit einem Rollenwechsel verbunden. Personen, die Erfahrungen mit einer oder mehreren Erkrankungen einbringen können, werden als Expert:innen mit Krankheits- und Versorgungserfahrungen wahrgenommen, die einen Beitrag zum Forschungsprojekt leisten. Darüber hinaus eröffnen Patient:innen zum Beispiel als Vertretende organisierter Strukturen wie der Selbsthilfe oder Patient:innenorganisationen Zugänge zu weiteren Erfahrungen und Netzwerken. Diese Patient:innen(vertretenden) werden nicht beforscht oder befragt. Sie sind ein Teil des Forschungsteams und forschen mit. In der englischen Definition für PPI, die vom National Institute for Health Research (NIHR) herangezogen wird, heißt es daher: „Research being carried out ‚with‘ or ‚by‘ members of the public rather than ‚to‘, ‚about‘ or ‚for‘ them“ [4].

In Deutschland hat sich ein Feld mit unterschiedlichen Begrifflichkeiten und eigenen Definitionen entwickelt. Patient:innenbeteiligung wird, vor allem, wenn es um aktive Mitwirkung geht, der partizipativen Forschung zugeordnet. Der wissenschaftliche Ansatz der partizipativen Gesundheitsforschung hebt die maximale Mitgestaltung am gesamten Forschungsprozess hervor [5]. Der partizipativen Versorgungsforschung hingegen geht es um das Einbringen von Expertise in möglichst vielen Phasen im Forschungsprozess [2].

Patient:innen bietet sich eine Vielzahl von Möglichkeiten, aktiv im Forschungsprozess mitzuwirken. Dabei kann die Beteiligung auf unterschiedlichen Stufen oder Intensitäten stattfinden. In Beiräten oder Panels beraten Patient:innen vor dem Hintergrund ihrer Erfahrungen die Forschenden. Sie können aber auch als Teil des Forschungsteams mit Wissenschaftler:innen unter Anerkennung der verschiedenen Expertisen gleichberechtigt mitarbeiten. Auch die Steuerung von Forschungsprojekten ist möglich, wenn Patient:innenorganisationen oder Interessenverbände die Projektleitung übernehmen und die Projekte selbst oder mit der Unterstützung von Wissenschaftler:innen durchführen.

Strukturell lassen sich so die Stufen der Beteiligung differenzieren in Beratung, Zusammenarbeit und Steuerung. Die Einordnung in Stufen ist eine Adaption der Leiter der Bürger:innenbeteiligung von Sherry Arnstein, die 1969 erstmals und im demokratischen Kontext verschiedene Beteiligungsgrade definiert hat [6]. Verschiedene Stufenkonzepte der partizipativen Forschung orientieren sich an dieser Grundlage [5, 7]. Die Beteiligung von Patient:innen kann in den unterschiedlichen Stufen oder Intensitäten im gesamten Projektverlauf stattfinden – von der Themenfindung bis hin zur Dissemination.

Im Folgenden werden 3 zentrale Aspekte näher erläutert, die verdeutlichen, wie Forschung relevant, passgenau und zugänglich für und mit Patient:innen gestaltet werden kann.

### Merke.

Forschung wird patient:innenzentriert, wenn sie Fragen beantwortet, die für Patient:innen relevant sind.

Patient:innenzentrierung stellt sicher, dass Forschung für Patient:innen bedeutsam ist. Jedoch werden Forschungsthemen häufig von Wissenschaftler:innen oder Förderinstitutionen festgelegt, ohne die Menschen einzubeziehen, die direkt von einer Erkrankung oder einem Versorgungsproblem betroffen sind. Um die Patient:innenzentrierung in dieser frühen Phase im Forschungsprozess zu fördern, gibt es unterschiedliche Ansätze.

Die James Lind Alliance ist eine Initiative aus England, die vor mehr als 20 Jahren damit begonnen hat, Forschungsthemen von Patient:innen, Angehörigen und Fachkräften im Gesundheitswesen zu identifizieren und priorisieren [8]. In sog. Priority Setting Partnerships werden über Umfragen und Workshops Forschungsfragen gesammelt, zusammengefasst und auf ihre Relevanz hin geprüft. Abschließend wird eine Prioritätenliste erstellt, die veröffentlicht wird und in Forschungsagenden einfließt [8]. In Anlehnung an die James Lind Alliance sind auch in Deutschland Prioritätenlisten entstanden [9–12]. Nun gilt es, ermittelte Forschungsprioritäten systematisch zu untersuchen [13].

Einen direkteren Weg wählt das Institut für Qualität und Wirtschaftlichkeit im Gesundheitswesen (IQWiG). Im Rahmen des „ThemenCheck Medizin“ haben Bürger:innen die Möglichkeit, Forschungsfragen an die Wissenschaft zu stellen und Vorschläge für wissenschaftliche Bewertungen diagnostischer oder therapeutischer Verfahren einzureichen.^1^

**Abb. 1:**
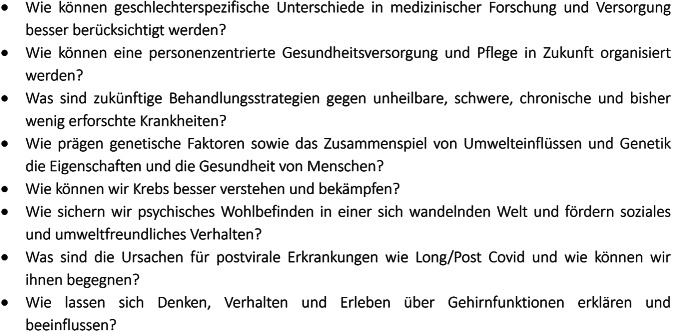
Fragen des Clusters Gesundheit aus der Umfrage #MeineFragefürdieWissenschaft im Jahr 2022 des Bundesministeriums für Bildung und Forschung (BMBF, seit 2025 Bundesministerium für Forschung, Technologie und Raumfahrt, BMFTR). (Wissenschaft im Dialog et al [14])

Mit einem ähnlichen Format hat auch das Bundesministerium für Bildung und Forschung (BMBF, seit 2025 Bundesministerium für Forschung, Technologie und Raumfahrt, BMFTR) im Rahmen des Wissenschaftsjahres 2022 mit #MeineFragefürdieWissenschaft eingeladen, Fragen zu einem wissenschaftlichen Thema zu stellen. Die fast 15.000 eingereichten Fragen zu allen Wissenschaftsbereichen wurden in einem partizipativen Prozess zunächst gebündelt und dann auf ihre Relevanz hin überprüft [14]. Die daraus thematisch gebündelten Fragen aus dem Cluster Gesundheit werden in Abb. 1 dargestellt.

Darüber hinaus hat das BMBF in einem Papier zur Partizipation Ansätze formuliert, wie die Patient:innenperspektive in die thematische Fokussierung und Ausgestaltung von Fördermaßnahmen einbezogen werden kann [15, 16].

Forschungsthemen können auch im direkten Austausch von Wissenschaftler:innen und Patient:innen entstehen, wenn frühzeitig Kontakt zu Selbsthilfe- oder Patient:innenorganisationen aufgenommen wird oder etablierte Kooperationen bestehen. Initiativen wie das Deutsche Zentrum für Psychische Gesundheit [17] oder auch die universitären Krebszentren [18] haben ihre Kooperationsstrukturen detailliert beschrieben. Auch bilden Patient:innenorganisationen eigene Strukturen aus [19]. Sie sind die Grundlage dafür, dass Forschungsprojekte schon während der Antragsphase abgestimmt werden und Entscheidungen gemeinsam getroffen werden können.

### Merke.

Forschung wird patient:innenzentriert, wenn Prozesse für teilnehmende Patient:innen passgenau sind.

Mittlerweile fordern immer mehr Forschungsförderer wie das BMFTR, der Innovationsfonds des Gemeinsamen Bundesausschusses (G-BA) oder die Deutsche Krebshilfe (DKH), Patient:innen in allen Phasen des Forschungsprozesses zu berücksichtigen. Die Antragsvorlagen umfassen daher Gliederungspunkte zur Patient:innenbeteiligung und die Antragstellenden beschreiben ihre Ansätze und Absichtserklärungen.

Im Rahmen von Förderentscheidungen bewerten Wissenschaftler:innen als Gutachter:innen die Voraussetzungen für eine erfolgreiche Projektdurchführung, die wissenschaftliche Qualität des Vorhabens und den zu erwartenden Erkenntnisgewinn. Für eine patient:innenzentrierte Forschung reicht diese wissenschaftsfokussierte Begutachtung jedoch nicht aus. Ergänzend müssen auch die Passgenauigkeit des Forschungsziels, die Angemessenheit für Teilnehmende sowie die Einbindung der Patient:innenperspektive berücksichtigt werden. Dafür ist es notwendig, Patient:innen als Gutachter:innen in Entscheidungsgremien zu beteiligen – wie es etwa beim BMFTR oder der DKH bereits der Fall ist. Indem sie dort ihre Erfahrungsperspektive in die Bewertung einbringen, lässt sich die Patient:innenzentrierung von Gesundheits- und Versorgungsforschung stärken und besser sicherstellen.

Doch auch nach der Förderentscheidung und während der Projektdurchführung sollte immer wieder die Passgenauigkeit der Abläufe für teilnehmende Patient:innen reflektiert werden. Daher ist ein weiterer zentraler Aspekt der Patient:innenzentrierung in der Forschung nach der erfolgreichen Antragstellung die Mitgestaltung bei der Durchführung von Forschungsprojekten. Immer häufiger werden Projekte durch Beiräte begleitet, die beratend oder teilweise auch kollaborativ in die Forschung eingebunden sind. Forschende bringen ihre wissenschaftliche Perspektive ein, während Patient:innen ihre Erfahrungen und Lebensrealitäten einbringen. Wie sich solche Beiräte im Projektverlauf und auch darüber hinaus etablieren können, wurde bereits dokumentiert [20, 21].

Darüber hinaus haben sich weitere Kooperationen ausgebildet, die zum Beispiel über die jeweiligen Webseiten der Forschungsverbünde oder -einrichtungen auffindbar sind. Exemplarisch sei hier auf die Webseiten des Neuro-Beirats der Universität Oldenburg^2^, den Bürger:innen- und Patient:innenbeirat des Deutschen Zentrums für Diabetesforschung (DZD)^3^ sowie den Forschungsbeirat der Palliativmedizin am Universitätsklinikum Erlangen^4^ verwiesen.

Während zunächst vor allem die Informationen der englischen Initiativen maßgeblich für die Ausgestaltung der Patient:innenbeteiligung waren [4], gibt es nun eine immer breitere Informationsbasis in Deutschland. Einen guten Überblick über die verschiedenen Formen der Zusammenarbeit haben Farin-Glattacker et al. [7] in Form einer Matrix gegeben. Es wurden Praxisbeispiele gesammelt [22] und unter Mitwirkung von Patient:innenvertretungen Leitfäden erstellt [23, 24]. Zudem werden Beteiligungsansätze in verschiedenen Forschungssettings in den Blick genommen [25, 26]. Dennoch bedarf es zusätzlicher Schulungen [27]. Dies liegt auch daran, dass Beteiligungsprozesse flexibel gestaltet werden müssen. Bestehende Erfahrungen bieten Orientierung, sie lassen sich jedoch nicht unmittelbar auf andere Projekte übertragen. Deshalb müssen zentrale Fragen in jedem Projekt neu beantwortet werden. Zu ihnen gehören:

- Mit welchem Ziel werden Patient:innen einbezogen?
- Wieviel Flexibilität kann in dem Projektantrag ermöglicht werden?
- Welchen Mehrwert können Patient:innen von der Beteiligung erwarten?
- Nach welchen Kriterien sollten Betroffene ausgewählt werden?
- Wie viele Treffen sind für die verschiedenen beteiligten Gruppen notwendig und realisierbar?
- Welche Art(en) der Kompensation sind anwendbar und für die Patient:innen angemessen?
- Welche Rahmenbedingungen sind im jeweiligen Beteiligungsprozess für die verschiedenen Gruppen erforderlich?
- Bedarf es zusätzlicher Ressourcen, wie z. B. Moderation, für den gemeinsamen Austausch oder Trainings für Forschende oder Patient:innen?
- Welche Prozessschritte müssen wie vorbereitet werden, damit Betroffene sinnvoll beteiligt werden können?
- Welche Qualitätsanforderungen gibt es an Patient:innenbeteiligung und werden diese erreicht?

### Merke.

Forschung wird patient:innenzentriert, wenn auch Patient:innen die Ergebnisse diskutieren können.

Open Science, also Forschungsergebnisse, Daten, Methoden und Publikationen offen zugänglich zu machen, wird zunehmend eingefordert, um Transparenz, Zusammenarbeit und gesellschaftliche Wirkung wissenschaftlicher Arbeit zu stärken [28]. Für Patient:innenbeteiligung reicht Open Science jedoch nicht aus. Die Projektergebnisse müssen zusätzlich so aufbereitet werden, dass sie auch für wissenschaftliche Laien verständlich sind.

Im Zuge von Open Science werden zunehmend in frei zugänglichen Zeitschriften und mit Verweis auf die Verfügbarkeit der Studie zugrundeliegende Daten publiziert. Bei Veröffentlichungen in englischsprachigen, frei verfügbaren Zeitschriften bleibt jedoch für viele Bürger:innen in Deutschland eine zweifache Sprachbarriere bestehen. Neben der englischen Sprache ist es auch die Fachsprache, die Verständnisbarrieren schafft. Immer mehr Fachzeitschriften erwarten daher laienverständliche oder grafische Zusammenfassungen. Ein Beispiel sind die Webseiten des Cochrane Netzwerkes. Über Cochrane Kompakt^5^ werden Informationen zu durchgeführten Übersichtsarbeiten verständlich aufbereitet und in mehreren Sprachen verfügbar gemacht, um eine breite Zugänglichkeit für Bürger:innen zu gewährleisten.

Neben der schriftlichen Kommunikation fördern auch Fachkongresse den Austausch. Veranstaltende laden vermehrt Bürger:innen oder Patient:innen ein. Damit diese Teilhabe jedoch wirksam ist und für alle Personen einen Mehrwert bringt, müssen die Formate an die Zielgruppen und deren Bedürfnisse angepasst werden. Dazu gehört beispielsweise, den Zugang durch geringe Gebühren oder kostenfreie Angebote niedrigschwellig zu gestalten, Sessions klar auszuweisen, die für Bürger:innen besonders relevant und verständlich sind, sowie eigene Veranstaltungsformate anzubieten, die sich explizit an diese Gruppen richten. Dies zeigte sich beispielsweise beim Deutschen Kongress für Versorgungsforschung (DKVF) 2025 in Hamburg, wo mit einem Patient:innen- und Bürger:innentag am 23.09.2025 patient:innenrelevante Themen zunehmend sichtbar gemacht, für diese Zielgruppen zugänglich aufbereitet sowie deren aktive Einbindung gefördert wurden.

Darüber hinaus wird Wissenschaftskommunikation und eine gezielte Kommunikation zunehmend relevant, die darauf abzielt, komplexe, wissenschaftliche Inhalte verständlich an Bürger:innen zu vermitteln. Sie setzt auf laiengerechte Informationsformate wie Broschüren, Podcasts oder Webseiten, die Forschungsergebnisse allgemeinverständlich erklären. Auch Dialogveranstaltungen, die einen Austausch zwischen Wissenschaftler:innen und Patient:innen ermöglichen, sind wichtig. Um breitere Zielgruppen zu erreichen, können und sollten nach Möglichkeit auch soziale Medien eingesetzt werden.

## Patient:innenzentrierte Forschung am Universitären Cancer Center Hamburg

Patient:innenbeteiligung entwickelt sich insbesondere in der onkologischen Forschung mit hoher Dynamik. Die Nationale Dekade gegen Krebs hat im Jahr 2022 einen Schwerpunkt auf Patient:innenpartizipation gesetzt und damit die Entwicklung der Strukturen weiter gestärkt. In diesem Rahmen wurde auch die „Allianz für Patientenbeteiligung in der Krebsforschung“ ins Leben gerufen, um diesen Aspekt in Deutschland langfristig als einen neuen Standard zu etablieren. Hierzu wurden Prinzipien für eine erfolgreiche Patient:innenbeteiligung in der Krebsforschung veröffentlicht [29], um eine Hilfestellung für die Praxis zu bieten.

Auf Seiten der Forschungsförderer nimmt die DKH eine Vorreiterrolle ein, indem sie in ihren Ausschreibungen und Begutachtungen Patient:innenbeteiligung einfordert. In den Universitären Krebszentren und den Nationalen Zentren für Tumorerkrankungen werden Patient:innenbeiräte und ergänzende Strukturen installiert [18]. Darüber hinaus unterstützen sehr engagierte Selbsthilfe- und Patient:innenorganisationen [30] den Prozess.

Im Folgenden werden exemplarisch die Strukturen für patient:innenzentrierte Forschung am Universitären Cancer Center Hamburg (UCC Hamburg) vorgestellt. Es soll aufgezeigt werden, wie Beteiligung in der Forschung praktisch umgesetzt werden kann und welche Elemente für eine nachhaltige Implementierung patient:innenzentrierter Forschungsprozesse förderlich sind. Die 3 zentralen Strukturen Patient:innenbeirat Forschung, Patient:innenbotschafter:innen und Patient:innenkongresse werden von einer Referentin und ihrem Team innerhalb des UCC Hamburg koordiniert und im Folgenden erläutert.

### Patient:innenbeirat Forschung

Der Patient:innenbeirat Forschung des UCC Hamburg besteht seit 2020 aus 12 Patient:innenvertretenden bzw. Eltern von erkrankten Kindern. Um vielfältige Erfahrungsperspektiven einzubringen, sind Personen aus verschiedenen Geschlechts‑, Alters- und Erkrankungsgruppen vertreten. Sie beraten Ärzt:innen und Wissenschaftler:innen hinsichtlich der strategischen Ausrichtung von Krebsforschung und -versorgung und prüfen die Ausrichtung am Patient:innennutzen sowie die Relevanz im Sinne patient:innenorientierter Fragestellungen und Bewertungskriterien.

Der Patient:innenbeirat tagt vierteljährlich und erhält die Möglichkeit, sich in speziellen Forschungsthemen fortzubilden. Darüber hinaus sind 2 Patient:innenvertretende Mitglieder im erweiterten Vorstand des UCC. Zusätzlich vertreten die Mitglieder den Beirat als Multiplikator:innen auf Veranstaltungen des Universitätsklinikums sowie extern und pflegen Kontakte zu regionalen und überregionalen Selbsthilfeorganisationen.

### Patient:innenbotschafter:innen

Das Patientenkompetenzzentrum Nord, „ONCOlleg“, bietet seit 2022 in Kooperation zwischen den UCC Hamburg und Schleswig-Holstein eine strukturierte Ausbildung zu Patient:innenbotschafter:innen in der Onkologie an. Ziel ist es, ehemals an Krebs erkrankte Personen und ihre Angehörige zu befähigen, ihre Erfahrungen in Versorgungs- und Forschungsprojekten einzubringen. Die Qualifizierung umfasst 10 Präsenz- und Onlinemodule einschließlich des Besuchs der Universitätskliniken in Hamburg, Kiel und Lübeck. Sie ermöglicht somit einen niedrigschwelligen und unterstützten Zugang zur Beteiligung in Forschungsprojekten.

Nach erfolgreichem Abschluss steht den Teilnehmenden die freiwillige Mitwirkung in verschiedenen Projekten offen. Dabei können sie selbstständig auswählen, an welchen Themenfeldern und Projekten sie interessiert sind. Dadurch ist eine Beteiligung unter Berücksichtigung der individuellen Interessen, Erfahrungen und Kompetenzen gewährleistet. Sie wirken dann zum Beispiel in Patient:innenbeiräten mit und bringen die Patient:innenperspektive im Projektverlauf ein. Sie können in dieser Funktion mit den Wissenschafter:innen diskutieren, ob Forschungsthemen relevant und Abläufe passgenau sind oder die Ergebnisse interpretieren. Der kontinuierliche Erfahrungsaustausch zwischen den Patient:innenbotschafter:innen, den Koordinator:innen und den Forschenden erfolgt in der sog. BOA-Runde (Botschafter:innenaustausch). Hier haben Wissenschaftler:innen die Möglichkeit, ihre Projektvorhaben vorzustellen.

### Patient:innenkongresse

In seit 2023 jährlich durchgeführten und von der DKH unterstützten Patient:innenkongressen werden verschiedene patient:innenzentrierte Themen wie State-of-the-Art-Medizin, Lebensstil und Umfeld in den Blick genommen. Das Programm mit Vorträgen, Interviews und Workshops zu Therapie und Unterstützungsangeboten erarbeiten Forschende und Patient:innenvertretende gemeinsam. Im Mittelpunkt stehen Austausch und Vernetzung.

## Zukunftsperspektiven für patient:innenzentrierte Forschung

Patient:innenzentrierte Forschung etabliert sich auf verschiedenen Ebenen immer mehr in der Wissenschaft. Wesentliche Treiber dieser Entwicklung in Deutschland sind, wie bereits beschrieben, Forschungsfördernde sowie Selbsthilfe- und Patient:innenorganisationen.

Neben vielen positiven Erfahrungen in der Zusammenarbeit werden auch Herausforderungen berichtet, etwa eine mögliche Überforderung von Patient:innen, begrenzte zeitliche Ressourcen von Wissenschaftler:innen, eingeschränkte finanzielle Mittel und eine geringe Flexibilität innerhalb von Forschungsprojekten.

Dennoch führt die zunehmende Verbindlichkeit dazu, dass Wissenschaftler:innen die Umsetzung der Patient:innenbeteiligung stärker forcieren. So enthalten Projektanträge inzwischen Abschnitte zur Patient:innenbeteiligung. Es muss jedoch gleichzeitig sichergestellt werden, dass diese Abschnitte auch dazu führen, dass Patient:innen tatsächlich adäquat einbezogen werden und patient:innenzentrierte Forschung durchgeführt wird. Da sich das Feld erst in den vergangenen Jahren etabliert hat, fehlt es vielen erfahrenen Wissenschaftler:innen an Umsetzungswissen. Praxisnahe Leitfäden und Beispiele können sowohl Forschende als auch Patient:innen bei der Umsetzung unterstützen. Ergänzend muss Patient:innenbeteiligung vermehrt in die wissenschaftliche Ausbildung integriert werden – im Studium und während der Promotionsphase.

Mit zunehmender Umsetzung wird auch das Berichten über Patient:innenbeteiligung relevant. Die GRIPP2-Checkliste (Guidance for Reporting Involvement of Patients and the Public) ist ein international konsentierter und unter Mitwirkung von Patient:innen(vertretenden) entwickelter Leitfaden zur Verbesserung der Transparenz und Qualität der Berichterstattung über Patient:innenbeteiligung [31]. Sie soll Forschende dabei unterstützen, die Art und den Einfluss der Patient:innenbeteiligung klar und nachvollziehbar darzustellen. Dadurch wird ermöglicht, dass sowohl Wissenschaftler:innen als auch Patient:innen und weitere Stakeholder von vorherigen Erfahrungen lernen können.

Das zunehmende Berichten bildet zudem die Grundlage für eine weitere wissenschaftliche Auseinandersetzung mit Patient:innenbeteiligung. Auch wenn aus gesellschaftlicher Sicht ethische und moralische Argumente für Patient:innenbeteiligung sprechen, sollte diese kritisch reflektiert und evaluiert werden. Einige wichtige Fragen, die in Zukunft wissenschaftlich beantwortet werden sollten, sind:

- Wann bringt Patient:innenbeteiligung für alle Beteiligten einen Mehrwert?
- Wie lassen sich Patient:innen für Beteiligung gewinnen und passenden Projekten zuordnen?
- Welche Fortbildungsformate (z. B. Wissenschaftskommunikation, partizipative Forschungsansätze) sind notwendig und geeignet, um Wissenschaftler:innen bei der Umsetzung von Patient:innenbeteiligung zu unterstützen?
- Welche Rahmenbedingungen und Ressourcen sind erforderlich für Patient:innen, um sich in Forschungsprojekten zu beteiligen?
- Welche Beteiligungsformate sind in welcher Projektphase und mit welchen beteiligten Akteuren am besten geeignet?
- Wie kann die Qualität der Patient:innenbeteiligung – sowohl hinsichtlich Prozess als auch Outcome – aus verschiedenen Perspektiven (z. B. Wissenschaftler:innen und Patient:innen) projektübergreifend und methodisch fundiert beurteilt werden?
- Wie wirken sich die unterschiedlichen Beteiligungsformate auf die Zusammenarbeit und die Ergebnisse von Forschungsprojekten aus?
- Wie kann sichergestellt werden, dass Beteiligung wirksam ist und nicht nur zum Schein durchgeführt wird?
- Inwieweit kann die Beteiligung von Patient:innen die Implementierung von Forschungsergebnissen in die Praxis fördern?
- Welche Ressourcen (z. B. Kompensation für Patient:innen, Zeit der Wissenschaftler:innen) und welche Infrastruktur (z. B. organisatorische Prozesse) sind für eine nachhaltige Umsetzung patient:innenzentrierter Forschung erforderlich?

## Fazit

Der Einbezug von Informationen von Patient:innen in den Forschungsprozess ist ein zentraler Faktor für eine patient:innenzentrierte Forschung. Partizipative Ansätze, in denen Patient:innen als Expert:innen aktiv und gemeinsam mit Wissenschaftler:innen den Forschungsprozess gestalten, etablieren sich immer mehr in der Gesundheits- und Versorgungsforschung in Deutschland. Durch Beteiligung erfahren Patient:innen Wertschätzung ihrer Erfahrungen und Wissenschaftler:innen erhalten weitere Einblicke in ihr Forschungsthema von außen. Neben den entstehenden Strukturen und den zunehmend verfügbaren Informationen sollte auch das Berichten der Beteiligungsprozesse sowie deren kritische Reflexion gefördert werden. Dieses Wissen fördert Mehrwerte für Patient:innen, Wissenschaftler:innen und Forschungsprojekte und hilft, Herausforderungen bei der Umsetzung zu bewältigen.

### Infobox Begriffliche Einordnung von Patient:innenbeteiligung im Forschungskontext

Beteiligung kann sich im Kontext Forschung nicht nur auf Patient:innen beziehen. Gemeint sind mit dem Begriff „Patient:innen“ in diesem Beitrag sowohl Personen, die über eigene Krankheits- und Versorgungserfahrungen verfügen, sowie Vertretende organisierter Strukturen wie der Selbsthilfe oder Patient:innenorganisationen. Jedoch identifizieren sich nicht alle Personen als Patient:innen. Bürger:innen, Betroffene oder andere Bezeichnungen sind durchaus üblich. Zudem bilden Patient:innen häufig nur eine Perspektive auf die Gesundheit(sversorgung) ab. Weitere Stakeholder wie Angehörige, medizinische Fachkräfte oder Akteure im öffentlichen Gesundheitssystem können ergänzende Perspektiven einbringen und ebenfalls in Beteiligungsansätze integriert werden. In diesem Beitrag wird vorrangig der Begriff „Patient:innen“ verwendet, wobei auch andere relevante Personen(gruppen) mitzudenken sind.

## Footnotes

1 https://www.iqwig.de/sich-einbringen/themencheck-medizin/, Zugriff am 24.11.2025.

2 https://uol.de/neuromodulation/neuro-beirat, Zugriff am 24.11.2025.

3 https://www.dzd-ev.de/das-dzd/struktur-und-gremien/buergerinnen-und-patientinnenbeirat, Zugriff am 24.11.2025.

4 https://www.palliativmedizin.uk-erlangen.de/forschung/unterstuetzung-forschungsbeirat/, Zugriff am 24.11.2025.

5 https://www.cochrane.org/de/evidence, Zugriff am 24.11.2025.
